# Postphenomenological Study: Using Generative Knowing and Science Fiction for Fostering Speculative Reflection on AI-nudge Experience

**DOI:** 10.1007/s11948-025-00534-3

**Published:** 2025-05-14

**Authors:** Ahreum Lim, Aliki Nicolaides, Xiaoou Yang, Beshoy Morkos

**Affiliations:** 1 School for the Future of Innovation in Society, Arizona State University, PO Box 876002, Tempe, AZ 85287-6002 USA; 2 Department of Lifelong Education, Administration and Policy, College of Education, University of Georgia, U.S.A River’s Crossing 417, 850 College Station Rd, Athens, GA 30605 USA; 3 School of Environmental, Civil, Agricultural and Mechanical Engineering, College of Engineering, University of Georgia, USA STEM Research, Building 1 Rm 2036, 302 East Campus Road, Athens, GA 30602 USA; 4 Department of Mechanical Engineering, School of Engineering, Santa Clara University, 500 El Camino Real, CA 95053 Santa Clara, United States

**Keywords:** Co-inquiry, Participatory, Pedagogy, Science fiction, AI education, Postphenomenology

## Abstract

This study presents an evidence-based argument for integrating participatory inquiry practices into AI education, using science fiction films as a primary tool for examining human-technology relationships. Through a media-enhanced co-inquiry approach, facilitators and students first explore the entanglements of human-technology interactions before engaging with AI nudges—productivity prompts introduced during time-constrained, interdependent assembly tasks in an experimental setting. A postphenomenological analysis of focus group interview data reveals that students’ collective responses to AI nudges reflect the competitive pedagogical culture of engineering, often reinforcing rigid, task-driven adaptation. However, moments of attunement to material conditions suggest that speculative thinking can serve as a catalyst for renegotiating entrenched norms of engineering rationality. By facilitating the movement of concepts and generating productive friction, speculation disrupts dominant conceptualizations of AI that the engineering community often readily subscribes to. This study highlights the necessity of a cultural shift in engineering education—one that embraces speculative inquiry as a means of fostering sociotechnical reflection and reimagining human-technology relations.

## Introduction

This study responds to Martin et al. (2021)‘s call for fostering “a socio-technical orientation of engineering education *for* ethics” (p. 59, italics in original) that challenges the dominant epistemic culture in engineering education, often marginalizing social consideration (Cech, 2014; Riley, 2013). Encouraging a sociotechnical approach to engineering practice starts with fostering active engagement with non-engineering perspectives (Downey, 2015). Given the “changing conceptions of the socio-material grounds of agency and lived experience” (Suchman, 2007, p. 139) in human-artificial intelligence (AI) interaction, promoting a sociotechnical framework in AI education for engineering students is crucial (Burton et al., 2015; Langley, 2019; Mackenzie et al., 2024).

As education theorists and mechanical and civil engineering faculty, we offer an evidence-based case for incorporating generative co-inquiry practices into AI education. Our approach (Anoynmized) emphasizes speculative thinking as essential for continuous inquiry into sociotechnical complexity. Generative knowing theory highlights the facilitator’s role in modeling and material encounters as a catalyst for speculative reflection on everyday sociomaterial structure (Edwards & Fenwick, 2015). Generative co-inquiry design actively adopts feminist science technology studies (STS) insights that promote ecological awareness and relational thinking through speculation (Haraway, 2016; Puig de La Bellacasa, 2017). As an open-ended exploration of relationships shaping ontological foundations, speculation enables shifts in perspective beyond fixed identities, cultivating the capacity for sustained negotiation with complexity (Lim & Nicolaides, 2024).

The primary research question guiding this study is: Does a media-enhanced generative co-inquiry facilitate speculative reflection on AI among undergraduate civil and mechanical engineering students? We explore this through a postphenomenological study in a lab-based experiment where students complete an assembly task with AI-driven prompts called AI nudges (Yang et al, 2022). These nudges, designed to enhance productivity, can be benign or coercive (Sætra, 2019; Yeung, 2017), prompting students to reflect on AI’s roles. Generative knowing theory shapes our co-inquiry practice, using science fiction as a pivotal educational tool. The first author facilitates inquiry for the experimental group and lectures for the comparison group to determine whether integrating co-inquiry practices, engagement with science fiction media, and lab-constrained AI experiences promote speculative reflection in engineering students.

We propose that students aware of the specific material conditions of AI nudges can better identify underlying assumptions shaping their AI perceptions, prompting a critical examination of socio-cultural templates communicated through science fiction. Using postphenomenology, we analyze students’ AI-mediated experiences to articulate how AI use in lab setup influences their bodily-perceptual habits (Rosenberger, 2023). This analysis explores whether speculative thinking can be a critical counterpoint to the pervasive cultural emphasis on scientific rigor in engineering knowledge (Kant & Kerr, 2019, p. 696). Thus, we aim to illuminate its potential to catalyze a transformative effect on engineering culture, challenging traditional paradigms and encouraging a more expansive view of what engineering knowledge can encompass (Martin et al., 2021).

Our discussion unfolds as follows: First, as an interdisciplinary team, including members with perspectives external to engineering, we highlight key literature shaping our understanding of engineering culture and the integration of generative knowing theory as a complementary pedagogic method. Next, we overview science fiction’s use in engineering education for teaching sociotechnical complexity and briefly review the postphenomenology informing the AI-nudge experiment’s design, data collection, and analysis. The findings are analyzed through a postphenomenological lens. Finally, we examine this approach’s pedagogical implications.

## Related Literature

### Engineering Education Culture & Its Solutionist Norms 

The evolution of engineering knowledge, shaped by professionalization, has seen a shift in engineering ethics from professional loyalty to broader public responsibility, driven by reflexive engagements with past failures and an awareness of technology’s societal impacts (Mitcham, 2009). This shift has broadened engineering ethics to emphasize public good and scientific rigor amid a wider scientification of engineering knowledge (Kant & Kerr, 2019, p. 696). A cultural reverence for science or celebration of its “detached objectivity” (Haraway, 1988) has reinforced systematic problem-solving in engineering, characterized by rigorous decision-making and efficiency, or as engineering rationality (Picon, 2004). Engineering rationality is solidified through the pedagogical choice of using competition in educational settings (Secules, 2019, p. 206), implicitly circulating the ideal engineering conception that relegates social considerations, perpetuating a disengaged culture (Cech, 2014) that marginalizes public concerns in engineering education.

Against this backdrop, feminist scholars provide conceptual tools for reimagining engineering culture as malleable rather than rigid. This perspective, rooted in relational ontologies, highlights how collective subjectivity is not pre-given but emerges dynamically through specific arrangements of people, objects, and practices (Haraway, 2016). Conceptualizing collective “we” through arrangement foregrounds the question of inclusion and exclusion, necessitating a mode of attention characterizied by care—what is made visible, valued, or erased within these sociomaterial configurations (Puig de La Bellacasa, 2017). From this perspective, culture plays a crucial role in learning, functioning as a diffractive medium composed of material-conceptual resources that shape and generate divergence in how individuals perceive, navigate, and respond to the world—even within a shared collective horizon of perspectives (Hasse, 2020, p. 125).

In engineering education, this relational framing of culture offers a means to breach solutionist beliefs that confine problem-solving to technically bounded, engineering-exclusive domains. A participatory approach to problem definition, rooted in care, offers an alternative to conventional engineering practices (Nair & Bulleit, 2020). This shift can be pedagogically transformative, encouraging engineering students to interrogate how problems are framed and whose perspectives are included (Downey, 2015). By integrating diverse stakeholder perspectives, this approach broadens the conception of “we” as problem-solvers, fostering sociotechnical competence (Smith et al., 2021). This is pertinent in holistic AI education, which moves beyond viewing AI as a “collection of disconnected algorithms” (Langley, 2019, p. 9671). A notable example is Mackenzie et al.’s (2024) summer school, which fostered cross-disciplinary dialogues on AI’s ethical and societal implications. Such collaboration enhances engineers’ humility and allows them to think through AI’s complex societal impacts, encouraging a broader, interdisciplinary approach to these “wicked” challenges (p. 11). The cultivation of humility signals a heightened awareness of the importance of forming a “we” that extends beyond disciplinary boundaries to address wicked challenges.

### Science Fiction: A Bounded Imagination of Human-Technology Relationship

Science fiction is integral for exploring and constructing possible worlds, acting as a conduit for forming powerful connections beyond established frameworks (Haraway, 1991, p. 299). Thus, it serves as a world-making experiment and a means to challenge, negotiate, and extend the prevailing norms, foregrounding “how to be response-able” (Haraway, 2013, p. 12) or engaging with the world differently. In the human-technology interface, this inquiry into response-ability relates to design ethics. It creates a space where diverse visions of future design processes are negotiated, wherein designers and engineers explore “heterogeneous ways to explore the unknown” with digital materials, users, and stakeholders (Croon, 2022, p. 241). Science fiction’s immersive nature prioritizes drawing spectators, readers, or other perceivers into imaginative worlds, facilitating perspective-taking and exploration into the unknown (Pendleton-Jullian & Brown, 2018). It also prompts moral reasoning (e.g., “Is it right to act that way?”), self-reflection (e.g., “Would the character’s response align with my course of action?”), and imagination (e.g., “What would I do in such a situation?”).

A cautious and critical engagement is necessary. STS scholarship emphasizes science fiction as a futurity narrative shaped by collective hope and fears (Jasanoff, 2015), serving as “both a critique of the present and a utopian anticipation of an improved life for humans” (Verschraegen et al., 2017, p. 10). Science fiction functions as a “mental template” (Ryan, 2006, p. 647) to navigate sociotechnical imaginaries bounded by the beliefs, obligations, and desires storytellers communicate. Despite its speculative, imaginative nature, science fiction remains anchored on the sovereignty of current institutions and norms, and writing about the imaginative world is shaped by daily life (Blackford, 2017), communicating primitive fantasies. For instance, the dominant aesthetics of robots in science fiction, including “its gleaming metallic and white plastic surface,” romanticize the future as “new, clean, and sparkling,” ostracizing diversity (Sparrow, 2020, p. 544). The pattern of hyper-sexualized, innocent characterization of AI in science fiction (Yee, 2017) underscores critical engagement with social norms embedded in these narratives. Science fiction often embeds such AI fetishism, “idealizing AI as perfections and perfectors of humans and human conditions” (Fuchs, 2022, p. 150). This view treats technological improvement as a prerequisite for social enhancement, potentially obscuring racially and socially biased cultural frameworks.

Science fiction intersects with STS perspectives and engineering ethics education in significant ways. Using science fiction as “multimodal case studies” enriches teaching engineering ethics (Hitt & Lennerfors, 2022, p. 44) by catalyzing moral imagination through multisensorial, context-rich narratives. Burton et al. (2015) demonstrate that such films create an immersive setting wherein computer science engineering students can learn ethical complexity non-threateningly. Additionally, they encourage engineers to expand the scope of design consideration by facilitating imaginative processes based on radical connections beyond established relationality frameworks. Its bounded imagination, shaped by social template, allows engineers to identify and interrogate how social desires for technological advancement is described in the film and fosters the reflection on the sociotechnical effect of emerging technologies. Therefore, it can be used to explore ethical debates surrounding emerging technologies (Delgado et al., 2012). Yet, effectively leveraging science fiction films in engineering ethics education depends on the educator’s capacity to create a dialogic space and integrate theoretical concepts (Teays, 2017).

To integrate these theoretical concepts, it is insightful to consider cultural critiques and discuss how science fiction portrays a spectrum of human-automation relationships (Visser et al., 2018). These narratives challenge automation’s traditional “thingness,” revealing more complex interactions beyond mere functionality (Duncan et al., 2023). Films like *Her* (2013) and *Ex Machina* (2014) depict AI characters who transcend their roles as tools, suggesting a co-constitutive relationship with humans that reshapes our understanding of agency and subjectivity. These narratives highlight AI’s potential to participate in relationships beyond their programming, demonstrating how technology might evolve into truly relational partners. For instance, in *Her* (2013), AI assistant Samantha’s relationship with its user, Theodore, is integral to his emotional development. Similarly, *Ex Machina* (2014) also speculates on human-AI interdependencies by conceptualizing AI’s creative potential beyond its programming. In the scene where Nathan (Ava’s developer) and Caleb (the male protagonist) converse before Jackson Pollock’s painting, Nathan compares Pollock’s improvisational painting with computing: “though requiring an artist’s hand, it can only reach total abstraction when it defies knowability” (Jacobson, 2016, p. 28). This scene communicates that AI can relate to humans in incalculable, undecipherable terms beyond the predefined roles like personal assistants. These stories contrast with the descriptions in *Norma Rae* (1979), which situates human-technology interactions within a broader context of labor exploitation, depicting technology as an element to resist through unionization (Taylor & Provitera, 2011).

### Postphenomenology: Critical AI Nudge Studies

Addressing AI challenges by destabilizing its “thingness” through situational approaches examines shifting sociomaterial agency, essential for recalibrating the critical engagement in increasing human-AI interdependencies (Suchman, 2007, 2023). Emerging technologies’ subtle yet sophisticated influence on sociomaterial structure is well articulated in the case of persuasive technologies. They contribute to the “technological milieu” (Aydin et al., 2019, p. 337), wherein human practices are enveloped, making critical engagement with the technological influence challenging. Identifying and interrogating bodily-perceptual habituation attached to a dominant technology use has critical value (Rosenberger, 2023). It foregrounds technological artifacts as active mediators of human experience (Verbeek, 2008), highlighting how these artifacts shape perception and action and reinforce specific repertoires that solidify social and cultural practices (Hasse, 2015). Focusing on micro-scale mediations of technology in human-world relations (Rosenberger & Verbeek, 2015) enables inquiry into the often-invisibly embedded social norms and scripts afforded by material structures.

AI nudge works as a conceptual figuration tool describing this hidden yet powerful intentionality of persuasive technologies through behavioral interventions (Caraban et al., 2019; Sætra, 2019; Yeung, 2017). Although framed as enhancing well-being, these nudges introduce ethical complexities, which raises concerns about autonomy, as they risk becoming covert mechanisms of influence that integrate seamlessly into human decision-making processes (Sætra, 2019). Even when consent is emphasized as a safeguard, it neglects how AI nudges function as “soft mechanisms of surveillant control” (Yeung, 2017, p. 129), subtly guiding behavior in difficult-to-detect ways. Framing the mixed intentionality of persuasive technologies through nudge design principles strategically exploits AI’s conceptual vagueness as a floating signifier—a term that appears to have a definite meaning yet broadly interpretable (Suchman, 2023, p. 3), befitting the postphenomenology’s approach to exploring micro-scale interaction between human and technology.

From a pedagogical perspective, this dynamic presents an opportunity for engineering ethics education to promote reflective engagement. Since AI nudges shape action and perception (Aagaard, 2017), educators can leverage postphenomenology to identify pivotal moments where students can interrogate their relationship with AI-mediated decision-making. Encouraging students to examine how technological mediations structure their perceptions and choices creates openings for reimagining agency beyond the assumptions embedded in automated systems. Consequently, postphenomenology helps foster ethical awareness and develop more nuanced engagements with AI-augmented environments.

### Holistic Approach to Reflection: Generative Knowing

Engineering educators use inquiry-based learning to promote meta-cognitive and reflective skills among students (National Research Council, 2004). As such, conventional inquiry-based training used in both engineering classrooms and labs emphasizes problem-driven thinking, encouraging students to engage self-directedly with learning materials in the classroom (Buch & Wolff, 2000) and acquire tacit knowledge integral to scientific knowledge production (Domin, 1999). However, this practice necessitates a more cautious approach as an overly simplified application may overlook critical opportunities to cultivate students’ epistemological reflexivity, awareness of its social relevance, and personal growth—dimensions essential to the holistic development of a scientific mindset (Huber, 2019).

Several cases explicitly highlight the holistic approach to reflection in engineering education. For instance, integrating the reflective practice on epistemological frameworks shaping how individuals link engineering practice and its societal impact (Burton et al., 2012) can transform problem framing in engineering education, reducing complex issues into simplified “problems of expected obedience,” (Riley, 2003, p. 139). A notable example is a Ph.D course for early-career scholars that fosters reflection on daily scientific research practices within the broader context of science-society expectations (Hesjedal et al., 2020). This design encouraged students to examine gaps between their daily practice theories-in-use related and espoused theory such as social policies, enhancing their sociotechnical awareness. Mackenzie et al. (2024) found that cross-disciplinary discussions on solutions to AI’s complex societal impacts across engineering, activism, philosophy, and business allowed engineers to revisit their solutionist beliefs and incorporate diverse perspectives.

A core principle of generative knowing aligns with the holistic application of theories of inquiry, viewing knowledge as inherently experiential, evolving through continuous interaction with the environment (Dewey, 1938/2015, p. 82). This perspective challenges the traditional separation of inner subjective and outer objective realms, emphasizing their intertwined nature and the constant negotiation constituting the relational ontology (Haraway, 2016). In this framework, knowing is always inherently active, always in action. The transformation of dormant knowing-in-action—be it presentational (artistic), propositional (theoretical), or practical (applicable) knowing (Heron, 1996)—is ironically catalyzed by being comfortable with the unknown. This staying with trouble (Haraway, 2016) is a resistant move to the socialized habit of mind that seeks, pursues, and attaches meaning to experience hastily. This perseverance of knowing-in-action thus translates into staying in a co-constitutive term with the experience of material encounters instead of imposing a reflective structure onto it (Fenwick, 2003). This continuously co-emergent and co-configured relationship between learner and environment creates a generative space for narrating and reinterpreting the experience with the material world. These encounters challenge fixed notions of certainty, offering opportunities to experimentally fabricate realities, “which are attached, gathered and negotiated as an ensemble of meanings, mattering, objects, subjects, human, non-human (Edwards & Fenwick, 2015, p. 1392).”

Generative knowing posits that facilitator-modeled co-inquiry promote knowing-in-action as a skill (Schön, 1992; Yorks & Kasl, 2002). When facilitators participate as “full participants,” they engage equally with other students with an advanced knowledge of the process (Yorks & Kasl, 2002). This approach allows students to articulate their thoughts in a dialogic space and to “move back and forth” between diverse interpretations (Schön, 1992, p. 133). The equal partnership between student and facilitator comes from the facilitators’ humble acknowledgment of their knowledge limit. Generative knowing suggests three key strategies for “co-creation with the unknown” (p. 24): through structure (designed constraints), embracing diverse learning forms (spacious freedom), and scaffolding engagement with the unknowable (thriving complexity). This approach depends on facilitators demonstrating epistemic humility, which involves managing participants’ comfort with uncertainty and transforming perspective on everyday practices and mundane objects, revealing how they are shaped (Edwards & Fenwick, 2015).

## Method

Our postphenomenology study (Rosenberger, 2023; Rosenberger & Verbeek, 2015; Verbeek, 2005) involves a lab-based experiment where engineering students participate in an interdependent assembly task with AI nudges. Ten teams of three were formed, and participants were informed that AI nudges for better productivity would be signaled through a restaurant pager. Before the experiment, participants attended either a co-inquiry practice or a lecture-based session, utilizing science fiction portrayal of human-AI interactions as key educational material. We theorize that students attuned to AI nudge’s material specificity can better recognize their knowing-in-action, enabling them to use different perspectives from science fiction to shape and question their experiences. This is a critical point for engineering ethics educators to intervene, encouraging students to recognize their knowledge limit and cultivating curiosity and wonder about the sociotechnical complexity of AI-an entry point for challenging solutionist approaches to AI in engineering (Downey, 2015; Mackenzie et al., 2024; Lim & Nicolaides, 2024). This leads to our main research questions:

1. How do AI signals mediate engineering students’ perceptions during a time-constrained, interdependent group assembly task?
2. What habitual ways of thinking and acting are revealed in engineering students’ responses to AI-mediated experiences?
3. Do science fiction narratives related to AI technologies influence the students’ perception of AI-mediated experience? If so, how?

### Experimental Set-Up

The experiment involved teams of three working on an interdependent assembly task with 37 components and 19 procedures, lasting between 20 and 30 min. Task distribution across workstations was uneven to simulate a real assembly line environment and encourage dynamic workflow adjustments. Work-in-progress (WIP) zones were used to gauge production rates and trigger tailored nudges based on team progress. Nudge signals are delivered via a restaurant pager, with three distinct nudge types—audio, visual, and haptic. Haptic nudges aimed at individual awareness, while audio and visual nudges extended to a shared domain, allowing others at adjacent workstations to perceive them (Yang et al., 2022).

The study employed experimental deception to underscore AI’s relational and contingent. Participants believed they were interacting with AI-managed systems when, in reality, the nudges were manually controlled. This approach strategically utilized AI’s conceptual vagueness as a floating signifier (Suchman, 2023, p. 3), allowing an investigative possibility into the nuanced appreciation of the AI nudge’s effects. We aimed to explore participants’ expectations for AI and their perception of AI nudges within a deliberately constructed sociomaterial context, both key to their conception of AI. This experimental deception is reviewed and approved by the external Institutional Review Board (IRB Protocol Number: Project00005092).

In our study, the facilitator’s role varied significantly between the experimental and comparison groups. In the experimental group, the facilitator (the first author) participated as a full participant, explicitly saying, “I am also here to learn with you.” The facilitator used prompts to guide discussions and share non-engineering perspectives to enhance learning collaboratively. After viewing film scenes, she encouraged participants to share initial reactions and reflect on their perceptions, including questioning underlying assumptions in their reasoning. When a participant critically analyzed technology portrayals, she encouraged further using the concept of multistability.

However, the facilitator assumed a traditional lecturer role in the comparison group. She used a scene from *Her* where AI assistant Samantha discusses Theodore’s divorce to introduce Bernard Stiegler’s concept of technicity—highlighting the philosophical notion that humans cannot exist apart from technology (see Appendix 1 for detailed scene descriptions and concepts).

**Fig. 1:**
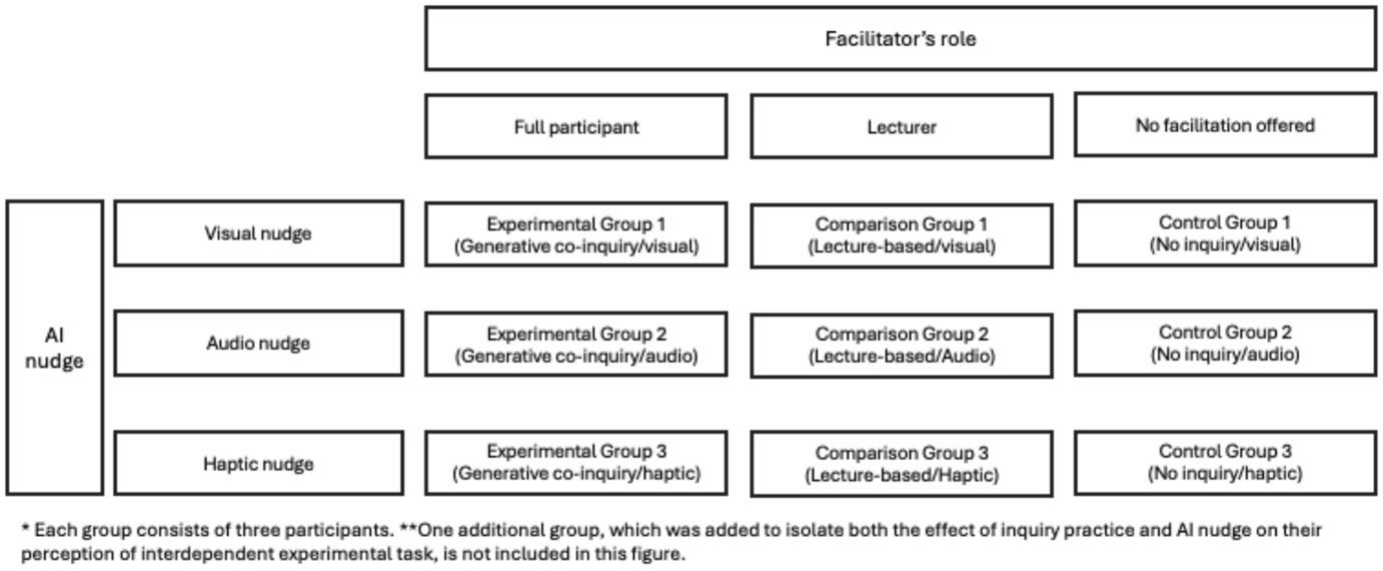
Experiment set up

The experimental and comparison groups varied in sensory nudges: visual (Group 1), audio (Group 2), and haptic (Group 3) (See Fig. 1). To isolate the facilitation effect, two control groups were established: three groups received the same types of nudges without any media-enhanced inquiry practice; another group received no nudges or engaged in inquiry, serving as a baseline for comparison.

### Participants

Thirty engineering students participated in the study. Three groups of three students received AI nudges without inquiry practice. Another group of three received neither AI nudges nor inquiry practice. This group was included to properly isolate the effect of AI nudges and inquiry practice and understand how students characterize an experimental task. Participants were recruited from a manufacturing design course taught by the fourth author, with consent forms stating that participation would not influence course grades. Most participants majored in mechanical engineering (66.7%), followed by civil engineering (13.3%), electrical engineering (10.0%), computer science engineering (3.3%), and no response (6.6%). Most participants identified themselves as juniors or students entering their junior year, with one freshman and six at a level above junior (Table 1).

**Table 1:** Demographics of the sample

	Experimental group (n = 9)			Comparison group (n = 9)			Control group (n = 12)		
Semesters taken	Average	Min, max	SD	Average	Min, max	SD	Average	Min, max	SD
	6.0	3.0,10.0	2.3	3.9	1.0,8.0	2.6	6.7	1.0,9.0	2.1

### Design of a Generative Co-inquiry

Generative knowing theory shapes a co-inquiry practice that leverages science fiction films to explore engineering’s impact on future work environments. This theory provides three key principles for creating a co-inquiry space, as detailed in Table 2. The authors used this framework to develop questions and cues that help students unpack sociotechnical interdependence depicted in the films.

**Table 2:** The feature of generative co-inquiry

Principles of designing generative inquiry space	Operationalization in this study
Designed constraints: fostering authentic dialogues through structured practice	First and second authors designed the facilitation cues script (for the details, see Lim & Nicolaides, 2023) Several inquiry practices include—surveying students’ immediate thoughts and feelings on the iOS alarm sound and a loading sign as a warm-up activity of reflecting on the daily experience of technological mediation (e.g., “How do you feel when you hear a notification sound or see a loading symbol on your screen?”)—introducing a collection of movie clips as learning material, yet playing a different role as full participant (experimental group) and lecturer (comparison group)
Spacious freedom: Utilizing story as a source of welcoming learning in any form: informational, instrumental, and transformational	Both groups were introduced to science fiction, which reflects societal concerns and technological possibilities and acts as a mental template to navigate the future, intertwined with the collective imaginations shaped by storytelling (Jasanoff, 2015; Ryan, 2006; Duncan et al., 2023)
Thriving Complexity: Providing support for methods of not stopping at the known but open to engaging with the unknowable (thriving complexity)	For the experimental group, the facilitator modeled epistemic humility by welcoming diverse perspectives (i.e., I am curious about your perspective), acknowledging her limit of knowledge (i.e., I am trained in adult education, so what you just said are new to me. Can you enlighten me?), and encouraging conversation (i.e., That speaks to how I view the movie clip)

### Data Collection and Analysis

We conducted focus group interviews and participant observations, following Aagaard et al.’s (2018) recommendation for employing Ihde’s postphenomenological approach. After experiencing AI nudges, students participated in focus groups, which were audio-recorded and transcribed. Each session lasted about 30 min, during which questions like “What stood out to you during the experiment?”, “Can you describe your nudging moment?”, and “What does nudging mean to you?” were posed. Concurrently, the first author documented participants’ physical responses during the experiments.

We adopted Adams and Thompson’s (2011) heuristics for a postphenomenological study in education, specifically (1) following the actors, (2) looking into the experiential structure of human-technology relations, and (3) studying breakdowns. Following the actors starts with the description of “what is being mobilized (knowledge, beliefs, or actions) in the shifting spaces created by the interactions between actors” (p. 738). We compiled participants’ data bits and the first author’s observation notes concerning moments that appear meaningful to the participants and the researcher. This leads us to generate a phenomenological anecdote (van Manen, 2017) portraying the experiential event as participants may have experienced across all groups. This anecdote highlights challenges in performing the experimental task. It serves as an entry point to examine AI nudge-student relationship and how nudges amplify students’ interaction with each other (Table 3). Breakdowns refer to the case wherein the objects gain visibility momentarily, revealing surprising associations across all sociomaterial elements afforded in the experiment setup (Adams & Thompson, 2011). Among 30 students, only Nate (CG 3^1^) and Tai (EG 3) used science fiction to narrate their nudging experience and reflected on their sociomaterial setup, guiding our focus on these cases as a breakdown.

**Table 3:** Focus-group interview results: perceived role of nudging

Nudge as a stressor	Nudge as a reminder
“Constantly back in my mind, like big brother always watching.” (CG 3) “a manager who doesn’t know what they’re talking about” (CG 1) “a manager who never worked a day in his life tries to manage you, giving you eyes like, ‘you better be working.’ Just visually expressing disappointment.” (CG 1) “a person over my shoulder telling me to move faster” (EG 1) “Let me do it fast, so that I can avoid it.” (CG 3)	“getting onto you for not doing a good job almost… who is instead of a human, but a machine.” (EG 2) “It was a sense to wrap up.” (EG 2) “I was working so that I wouldn’t hear the buzz.” (EG 2) “It kind of reminds me like a message notification” (EG 3) “It was just affirmation of what I already knew” (EG 3) “That pacer to keep aware of what’s going on.” (EG 2) “So you got nudge, so you needed to be go quicker.” (CG 2)

## Findings

### Anecdote: Challenges Across all the Groups

“Now, you can turn to the three stations behind you. You can choose whichever station you want to go to.” With the researcher’s guidance, three engineering students turned to see the three stations set up behind them. Each station was carefully arranged, with five red boxes containing essential parts to assemble the power tools on the desks. The students exchanged quiet glances, each finding their place. Instructions at each station clarified their assigned tasks, each requiring varying assembly skills.

Initially, participants needed time to fully step into their assigned roles at each station. As they became immersed in the task, these roles started to solidify. Participants described their emerging roles differently: one as the “stage-setter” (1st station) (CG 2), another as the “smooth transitioner” (2nd station) (CG 3), and yet another as the “machinic screw-er” (3rd station) (C 2).

However, these roles brought challenges. At the first station, screws were a persistent problem, leading to frustration. Some participants raised their hands, informing on-site observer that “things [screws] ain’t going in.”(EG 2). At the second station, others wrestled to fit parts together. “A lot of those wires were just like all over the place,” one participant (CG 3) remarked. The third station encountered fewer issues, as their job was relatively straightforward—constant screwing. When asked about their overall impression of the task, some participants jokingly mentioned the burning pain in their forearms and how mind-numbing it felt.

Despite these hurdles, participants were committed to creating a consistent flow by assembling eight power tools. When asked about their motivation, they cited their understanding of engineering tasks, mentioning “optimization” or “standardization” as driving forces. Dan and Andy (CG 2) connected their assessment of the nudging situation to knowledge acquired from “the special topics class a week ago.” Dan elaborated:

“Because at the end of the day, …, in a manufacturing setting, if we’re not all working at the same rate, or working hard to get this final goal or product delivered, then we’re not getting paid, …, you might not have a job.”

In one instance, a participant from C2 voluntarily switched stations with another participant struggling to understand the process, resolving a bottleneck moment he had created. When asked why, he replied, “It was simple math at that point where the bottleneck was.” His quick decision and the lack of objection from others demonstrated the group’s goal of optimizing the process. This rationalized construction of the nudging situation influenced the participants’ perception of technological nudging (Table 3).

All participants associated a nudge either as a “stressor” or a “reminder” that pushed them to work faster. Those who associated nudge as a strong push for enhanced performance reacted adversely to the nudging signal from the pager. Others who perceived nudge as a simple reminder of their process showed less intensive response. Using these two themes as a guideline, we suggest two cases of breakdown: Nate and Tai.

### Case of a Breakdown: Nudge as a Big Brother

One metaphor used by Nate caught researchers’ attention. Nate described his interaction with the nudge device as stressful, comparing it to the persistent surveillance of “big brother.” He likened it to running competitively, where others’ presence pushes him to run harder than if he were alone:

It’s …like running, … when I run with somebody else. I feel like I’ve always got to like run at the top of my game although I got tired out sometimes, whereas if I’m running by myself, sometimes I’m… Oh man, I’m going a little tired. Maybe I can slow down.

Dana and Jim echoed this sentiment of competition during a focus group, with all three expressing that the experiment felt like a competition, a dynamic the researchers had not anticipated:

Dana: Somehow it felt like a competition.

Nate: Yeah, exactly.

Jim: We definitely felt that we are competing with another set.

Nate called this a “secondhand nudge,” where observing others’ work rates spurred him to work faster. His experiences highlight a collective learning dynamic influenced by AI nudges and peer interactions, suggesting that human–human interactions alongside human-technology relations can significantly shape technological experiences (Hasse, 2018; Ihde, 2010; Verbeek, 2005).

Nate compared the tension induced by AI nudges to *Ex Machina*, interpreting the nudges as a “negative relationship” and “source of stress.” His focus was less on the philosophical implications of technology, as suggested by the movie, and more on his personal strain and the observational pressure from the AI, reflecting on how AI nudges made him feel watched and pushed to his limits.

Most of the stuff that you covered beforehand, in terms of …, artificial intelligence. And I have some thoughts about that, how that connects to … humans, where it’s difficult talking about something that’s just, you know, haptic feedback. So … in the very beginning, the Ex Machina was talking about how he programs sexuality, because sexuality, I guess, invokes a sense of communication that is difficult … . It [nudge] had an interaction with us. And it forced us to form a relationship with that device. For some, it may be positive, some negative for me, like I said, it was a source of stress.

### Case of a Breakdown: Stereotyping AI

During the focus group interview, Tai from EG 3 shared her thoughts regarding sensorial contact during the experiment, which resonated with Rachel, another group member.

Tai: … if you had a woman walk in and tell me she was a robot and she was the one telling me like, ‘Tai, move quicker’

Rachel: Yeah, I was just thinking that too.

Tai: Yeah, that’s different from the thing in my pocket. … But I think it’s also just like a preconceived notion that I already have about AI and what I think it is because when you say AI I don’t think about a buzzer. …

Tai, like Nate, found her sensory interaction with the nudge device inconsistent with her anthropomorphic view of AI. However, a conversation with Rachel prompted her to reconsider her assumptions about AI. Tai’s reflection began with the sensory experience of the nudge, particularly recognizing its sound, which conflicted with her stereotypical views of AI. This sensory trigger made her question her preconceived notions of AI and explore how her immediate interactions influenced her understanding.

When asked, “What if the nudge comes from human rather than artificial intelligence?” the group discussed a possible scenario where they might feel frustrated and find it hard to respect the robots yet would still respect evaluations based on data and concrete facts, albeit with some doubt. Tai marked,

“...my first thought is that if humans controlled it, they (humans) try their best, but they’re going to be biased because we are human. So I feel like it can be very much opinionated too.”

Tai’s skepticism about AI nudging—triggered by the mechanical buzz that seemed primitive compared to humanoid AI—spurred a discussion on AI’s socially constructed aspects. Her probing of established AI concepts showed an increased awareness of her assumptions. Intrigued by the practical effects rather than AI’s theoretical representations, Tai avoided the “lure of anthropomorphism” (Suchman, 2023, p. 3), attesting to the difference from Nate. However, she did not thoroughly critique AI’s normative conception as depicted in science fiction or its underlying AI fetishism, which “idealizes the non-human as perfections and perfectors of humans and human conditions” (Fuchs, 2022, p. 150). Nevertheless, Tai’s views did not challenge the broader ideological portrayals of AI without bias. This idealized conception of non-human agents stopped short of addressing deeper socio-political structures, underpinning the current illusion of unbiased AI.

## Discussion

This study explored how engineering students perceive and interpret AI nudging within a generative space of inquiry, analyzed through a postphenomenological lens. Three key areas emerged for further discussion: (a) the collective process of learning technologically textured experiences within the epistemic culture of engineering education, (b) material perception as an integral point of mobilizing and inflecting knowing, and (c) the pedagogical implication for constructing a space to negotiate diverse AI perspectives.

This study’s primary finding is that AI nudge experiences are collectively perceived through implicit scripts within the socio-technical milieu, particularly shaped by engineering’s pedagogic culture (Picon, 2004; Secules, 2019). In the experimental setup with invitational qualities that allow students to adjust their capacities, AI nudge functioned as a context-stimulating technology (Coeckelbergh, 2020), orchestrating group dynamics that progressively foster a perceptually competitive environment. Students also reported optimizing the work process and adjusting capacities, shaped by their systematic approach to unexpected challenges rooted in disciplinary training. The goal of making the production rate consistent shows how engineers’ training constitutes their problem definition (Downey, 2015) and engineering rationality (Picon, 2004). More remarkable is the affective resonance among students who fear becoming the team’s weak link. Despite the frustrations, nudges still influence students. Instead of ignoring the nudges, students accelerate their processes to meet unrealistic demands, often resulting in physical pain. Coupled with the competitive microcosm within the experimental setup, students feel compelled to respond to the nudges rather than withdraw from the socio-technical system in which they actively participate. This echoes how persuasive technology intensifies the entanglement between humans and technology, a dynamic described as the “technological milieu” (Aydin et al., 2019, p. 337), which envelops human practices emerging from technology’s effects.

This passive acceptance of nudge as a stressor reinforces engineering education’s competitive cultural patterns, normalizing a winner-loser framework and ostracizing those deemed incompetent (Secules, 2019). In this cultural template, the nudge’s intended function as a benign prompt for productivity becomes a compelling shove (Sætra, 2019)—when combined with a system designed to deliver highly personalized prompts, the users’ responses become part of a feedback loop created by the nudge, making it difficult for users to disengage. This demonstrates how AI nudge experiences are collectively shaped by the broader social context in which students operate (Hasse, 2020)—specifically, the culture of engineering education.

The second finding highlights a nuanced difference between experimental and comparison groups in terms of integrating the anthropomorphic portrayal of AI in science fiction movies. Unlike most participants, only Tai and Nate integrated science fiction narratives in conceptualizing and articulating their encounters with AI nudges. They used the features depicted in the movies (i.e., communicative form for Nate, Human-like features for Tai) to describe their understanding of the AI nudge experience. While Nate referenced *Ex Machina* to narrate his AI nudge experience, his connection remained cursory, lacking critical engagement with the movie’s portrayal of human-technology interaction. Conversely, Tai actively reflected on the human-like features of AI depicted across movie clips and connected this to her material encounters with AI nudges. This contrast illustrates how science fiction or its narrative element serves as a toolbox for tracing and speculating on human-technology relations (Haraway, 2013), though their impact is uneven among students. Science fiction serves as a valuable cognitive resource (Hitt & Lennerfors, 2022), enabling students to navigate the complexities of their AI-influenced experiences through narrative. However, for students to develop deeper connections between science fiction and their technologically textured experiences, engineering educators’s skillful facilitation matters (Teays, 2017).

Nate and Tai also differed in terms of perceiving the material specificity of AI nudges. While Tai remained discerning in terms of her conception of AI based on her attunement to the material specificity of the AI nudge she experienced—a buzzing sound—while Nate made little mention of his sensory contact with the nudging device. Nate’s configuration of nudge as a big brother easily attached to the “lures of anthropomorphism” (Suchman, 2023, p. 3) that undergird the public discourse around AI, including science fiction movies which were used in the inquiry while Tai interrogated it. Further, her attunement to the material quality of experience mobilizes her into question the “stereotyped” AI. This shows how she is already “moving back and forth” between diverse conceptions of AI, implying a potential of noticing her knowing-in-action.

This leads to the third insight, addressing the nuanced role of speculative thinking in shaping students’ interpretations of AI nudging. In both breakdown cases, Tai and Nate’s perceptions of AI nudging were co-constructed through material and social interactions, yet with a nuanced difference. While Tai demonstrated an awareness of her preconceptions about AI, Nate did not. This awareness surfaced her previously unexamined assumptions about AI or her stereotypes, inviting her to engage in a cycle of inquiry that explores diverse interpretations of AI. Tai’s speculative thinking, invoked by the friction between her embodied response to AI nudges (micro-perception) and the broader conceptual frameworks from science fiction (macro-perception), reveals rich learning possibilities. Her interpretation of AI as a surveillance tool reflects her lived experience with the nudges, while her speculative “what-if” thinking provided her with “room for maneuver” (Aagaard, 2017, p. 527) to critically engage with the affective dimension of the AI experience. This engagement with speculative thinking highlights how the process of stabilizing technologically mediated experience is ripe for learning opportunities in which engineering ethics educators can intervene and facilitate deeper discussions around the boundaries of perception and imagination. Quite interesting is how her knowing-in-action did not reach the point of interrogating the normalized conception of romanticizing technological development as a social improvement.

Her case illustrates how destabilizing technologically mediated experiences offer valuable learning opportunities for engineering ethics educators. Educators can foster deep discussions about the romanticization of technology-driven systems by critically examining her “stereotyped” view of AI as more objective than humans by facilitating what representation, artifacts, or people motivated her knowing-in-action to attach to the fetishized AI network (Edwards & Fenwick, 2015). These discussions can develop into how such networked perspectives that are unknowingly prevalent turn oblivious to the socio-political forces shaping the development of AI. This open-ended ensemble of matter and meaning (Edwards & Fenwick, 2015), marked by its aporia, ironically becomes generative (Lim & Nicolaides, 2024).

This points to the value of cultivating half-baked knowledge (Dewey, 1938/2015) to expand students’ ethical and imaginative capacities within the structured epistemic culture of engineering. While a single intervention may not transform students’ perspectives, encouraging speculative interweaving of ideas allows students to explore and potentially reframe the culturally structured worlds shaping their situated knowledge. In an educational context that often prioritizes fully-formed, concrete knowledge, the concept of half knowledge—speculative, incomplete, and open-ended—can foster a broader and more ethical outlook. Tai’s organic integration of speculative thinking, reflecting on her AI-textured experience, hints at the transformative possibility that disrupts the epistemic and educational culture of engineering. While students’ collective learning process of AI nudge experiences reflects the competitive culture of engineering education—momentarily aligning them as rigid, unbreakable collectives—these material-conceptual entanglements that bind engineering students’ perception of AI as a collective phenomenon (Hasse, 2020) can be contested through speculative thinking. Speculation facilitates the travel of concepts, generating a productive friction that challenges the dominant conceptualization of AI to which the engineering community often readily subscribes.

As a pedagogical application of this finding, we recommend designing learning environments that support speculative inquiry to fully harness the epistemic and pedagogical potential of science fiction narratives and their engagement with possible worlds. A cross-disciplinary educational setting is particularly well-suited to cultivating such a speculative space—one in which engineering students can critically explore the varied spectrum of human-AI interaction in an open-ended manner, enriched by non-engineering perspectives. Science fiction narratives, in this context, serve as a productive heuristic for students to engage diffractively with diverse AI-human relations, challenging dominant technological imaginaries. This process can be further facilitated through the integration of participatory co-inquiry practice, generating diffractive entry points for both engineering and non-engineering students to engage with the sociotechnical complexities of real-world technological systems. Ultimately, such an approach encourages students to rethink their roles as engineers in shaping technology and to consider “how to be response-able” (Haraway, 2013).

## Conclusion

This study empirically examines how space construction of generative co-inquiry encourages speculative reflection among engineering students. By facilitating learning opportunities to continuously revisit routine engineering epistemologies through diverse speculative narratives and collective inquiry, this approach reflects the potential for shifting the epistemic and pedagogical culture of engineering. Although it may be optimistic to argue that a one-time intervention that deliberately incorporates science fiction based on feminist STS perspectives could create a cultural shift in engineering education, our evidence-based argument implies such a cultural shift may be conceivable at the micro-political level. Engineering ethics educators, by attuning to students’ thought processes and creating a space of inquiry that warmly embraces exploratory, half-baked ideas—even those that fall outside traditional engineering rationality—may play a critical role in encouraging this shift.

## Appendix 1

See Table 4.

**Table 4:** Science fiction characters and matching concepts

Movie	Scene	Philosophical Concepts/Main inquiry
Ex Machina (Garland, 2014)	Nathan (the creator of Ava, a female AI robot) explains his vision of computing through Jackson Pollock painting to Caleb (the male protagonist)	Heidegger’s technology (Enframing) The artistic process, as a form of technology, reveals the latent possibilities within the world, enabling these elements to be perceived and utilized as forms of art What does AI reveal in your life?
Her (Jones, 2013)	Samantha (AI assistant) helped Theodore (the male protagonist) remember the happy memory with his ex-wife, showing emotional support	Stiegler’s technicity Tool also invents human—humans are existing with the technology in a mutually transformative way (Duncan et al., 2023) What are the changes you experienced in using AI? How did it feel?
Norma Rae (Ritt, 1979)	Norma Rae (the female protagonist) shut down the machine in textile factory, as a sign of resistance, and stood up in the middle of workers	Simondon’s subjectification and technology Technology affects worker identity formation and vice versa Why do you think workers resist the technologically-augmented factory? What constitutes their sense of vulnerability?
Avengers: Age of Ultron (Whedon, 2015)	Tony Stark (the male protagonist) and Banner (supporting character) discussed the way to revive Jarvis	“Humanized” relationship with AI: Can AI become a friend? What defines friends and befriending with AI?
Wall-E (Stanton, 2008)	The secret AUTO (the authoritative robot) kept from the captain is revealed, and AUTO starts mutiny	“Dehumanized” relationship with AI: What are the anticipated case of AI working as a technical governance or surveillance? Why does it feel dehumanized?
Interstellar (Nolan, 2014)	TARS made an inappropriate joke about robot colony and the protagonist Cooper commands to lower the humor level	“Humanized” relationship with AI: Can humor or human emotions be codified? What other aspects of human-like features can be embedded in the technology?

## Footnotes

1 The groups will be referred to by their abbreviation for better readability. EG: Experimental Group (Co-inquiry), CG: Comparison Group (Lecture), C: Control Group, 1: visual nudge, 2: audio nudge, 3: haptic nudge. All the names are pseudonyms.

## References

[CR1] Aagaard, J., Friis, J. K. B., Sorenson, J., Tafdrup, O., & Hasse, C. (2018). *Postphenomenological methodologies: New ways in mediating techno-human relationships*. Rowman & Littlefield.

[CR2] Aagaard, J. (2017). Introducing postphenomenological research: A brief and selective sketch of phenomenological research methods. *International Journal of Qualitative Studies in Education,**30*(6), 519–533. 10.1080/09518398.2016.1263884

[CR3] Adams, C. A., & Thompson, T. L. (2011). Interviewing objects: Including educational technologies as qualitative research participants. *International Journal of Qualitative Studies in Education,**24*(6), 733–750. 10.1080/09518398.2010.529849

[CR4] Aydin, C., González Woge, M., & Verbeek, P.-P. (2019). Technological environmentality: Conceptualizing technology as a mediating milieu. *Philosophy & Technology,**32*(2), 321–338. 10.1007/s13347-018-0309-3

[CR5] Blackford, R. (2017). *Science fiction and the moral imagination: Visions, minds, ethics*. Springer.

[CR6] Buch, N. J., & Wolff, T. F. (2000). Classroom teaching through inquiry. *Journal of Professional Issues in Engineering Education and Practice,**126*(3), 105–109. 10.1061/(ASCE)1052-3928(2000)126:3(105)

[CR7] Burton, R., Schlemer, L., & Vanasupa, L. (2012). Transformational innovation: Reflections on how to foster it in engineering education systems. *International Journal of Engineering Education*. https://www.semanticscholar.org/paper/Transformational-Innovation%3A-Reflections-on-How-to-Burton-Schlemer/fde1d84abe433cb7cbd45b2461ea691b21832534

[CR8] Burton, E., Goldsmith, J., & Mattei, N. (2015). Teaching AI ethics using science fiction. *Papers from the 2015 AAAI workshop*. Association for the Advancement of Artificial Intelligence. https://aaai.org/papers/aaaiw-ws0083-15-10139/

[CR9] Caraban, A., Karapanos, E., Gonçalves, D., & Campos, P. (2019). 23 ways to nudge: A review of technology-mediated nudging in human-computer interaction. In *Proceedings of the 2019 CHI conference on human factors in computing systems* (pp. 1–15). 10.1145/3290605.3300733

[CR10] Cech, E. A. (2014). Culture of disengagement in engineering education? *Science, Technology, & Human Values,**39*(1), 42–72. 10.1177/0162243913504305

[CR11] Coeckelbergh, M. (2020). Technoperformances: Using metaphors from the performance arts for a postphenomenology and posthermeneutics of technology use. *AI & Society,**35*(3), 557–568. 10.1007/s00146-019-00926-7

[CR12] Croon, A. (2022). Thinking with care in human–computer interaction. *Feminist Theory,**23*(2), 232–246. 10.1177/14647001221082294

[CR14] Delgado, A., Rommetveit, K., Barceló, M., & Lemkow, L. (2012). Imagining high-tech bodies: Science fiction and the ethics of enhancement. *Science Communication,**34*(2), 200–240. 10.1177/1075547011408928

[CR70] de Visser, E. J., Pak, R., & Shaw, T. H. (2018). From ‘automation’ to ‘autonomy’: The importance of trust repair in human–machine interaction. *Ergonomics 61*(10), 1409-1427. 10.1080/00140139.2018.1457725 10.1080/00140139.2018.1457725 29578376

[CR15] Dewey, J. (2015). *Experience and education*. Free Press. (Original work published 1938)

[CR16] Domin, D. S. (1999). A review of laboratory instruction styles. *Journal of Chemical Education,**76*(4), 543. 10.1021/ed076p543

[CR17] Downey, G. L. (2015). PDS: Engineering as problem definition and solution. In *International perspectives on engineering education* (pp. 435–455). Springer. 10.1007/978-3-319-16169-3_21

[CR18] Duncan, P., Henry, C., & Molloy, M. (2023). Posthuman as genre. In M. Molloy, P. Duncan, & C. Henry (Eds.), *Screening the posthuman* (p. 0). Oxford University Press. 10.1093/oso/9780197538562.003.0002

[CR19] Edwards, R., & Fenwick, T. (2015). Critique and politics: A sociomaterialist intervention. *Educational Philosophy & Theory,**47*(13/14), 1385–1404. 10.1080/00131857.2014.930681

[CR20] Fenwick, T. (2003). Reclaiming and re-embodying experiential learning through complexity science. *Studies in the Education of Adults,**35*(2), 123–141. 10.1080/02660830.2003.11661478

[CR21] Fuchs, C. (2022). Robots and artificial intelligence (AI) in digital capitalism. In *Digital humanism* (pp. 111–154). Emerald Group Publishing Limited. 10.1108/978-1-80382-419-220221005

[CR22] Garland, A. (Director). (2014). *Ex Machina* [Film]. Film 4.

[CR23] Haraway, D. (2013). SF: Science fiction, speculative fabulation, string figures, so far. *Ada: A Journal of Gender, New Media, and Technology.*10.7264/N3KH0K81

[CR24] Haraway, D. (2016). *Staying with the trouble: Making Kin in the Chthulucene* (p. dup;9780822373780/1). Duke University Press. 10.1215/9780822373780

[CR25] Haraway, D. (1991). *The promises of monsters: A regenerative politics for inappropriate/d others*. Routledge.

[CR75] Haraway, D. (1988). Situated knowledges: The science question in feminism and the privilege of partial perspective. *Feminist Studies,**14*(3), 575-599. 10.2307/3178066

[CR27] Hasse, C. (2015). The material co-construction of hard science fiction and physics. *Cultural Studies of Science Education,**10*(4), 921–940. 10.1007/s11422-013-9547-y

[CR71] Hasse, C. (2018). Studying the telescopes of others: Towards a postphenomenological methodology of participant observation. In J. Aagaard, J. Kyrre Berg Friis, J. Sorenson, O. Tafdrup, & C. Hasse (Eds.), *Postphenomenological methodologies: New ways in mediating techno-human relationships* (pp. 241-259). Lexington Books.

[CR28] Hasse, C. (2020). *Posthumanist learning: What robots and cyborgs teach us about being ultra-social*. Routledge.

[CR29] Heron, J. (1996). *Co-operative inquiry: Research into the human condition*. (pp. 1–240). https://www.torrossa.com/en/resources/an/4913413

[CR30] Hesjedal, M. B., Åm, H., Sørensen, K. H., & Strand, R. (2020). Transforming scientists’ understanding of science-society relations. Stimulating double-loop learning when teaching RRI. *Science and Engineering Ethics,**26*(3), 1633–1653. 10.1007/s11948-020-00208-2 32180098 10.1007/s11948-020-00208-2 PMC7286945

[CR31] Hitt, S. J., & Lennerfors, T. T. (2022). Fictional film in engineering ethics education: With Miyazaki’s the wind rises as exemplar. *Science and Engineering Ethics,**28*(5), 44. 10.1007/s11948-022-00399-w 36098844 10.1007/s11948-022-00399-w PMC9470632

[CR32] Huber, L. (2019). Reflection. In H. A. Mieg (Ed.), *Inquiry-based learning – undergraduate research: The German multidisciplinary experience* (pp. 81–90). Springer. 10.1007/978-3-030-14223-0_8

[CR76] Ihde, D. (2010). *Technology and the lifeworld: From garden to earth*. Indiana University Press. (Original work published 1990)

[CR33] Jacobson, B. R. (2016). Ex Machina in the garden. *Film Quarterly,**69*(4), 23–34. 10.1525/fq.2016.69.4.23

[CR34] Jasanoff, S. (2015). One future imperfect: Science, technology, and the imaginations of modernity. In S. Jasanoff & S.-H. Kim (Eds.), *Dreamscapes of modernity: Sociotechnical imaginaries and the fabrication of power* (pp. 1–33). University of Chicago Press. 10.7208/9780226276663-001

[CR35] Jones, S. (Director). (2013). *Her* [Film]. Annapurna Pictures.

[CR36] Kant, V., & Kerr, E. (2019). Taking stock of engineering epistemology: Multidisciplinary perspectives. *Philosophy & Technology,**32*(4), 685–726. 10.1007/s13347-018-0331-5

[CR37] Langley, P. (2019). An integrative framework for artificial intelligence education. *Proceedings of the AAAI Conference on Artificial Intelligence*. 10.1609/aaai.v33i01.33019670

[CR74] Lim, A., & Nicolaides, A. (2023). A socio-material approach to professional learning for engineers in industry 4.0.: In response to the emergent human-machine interaction. In L. Formenti, A. Galimberti, & G. Del Negro (Eds.), *New seeds for a world to come. Policies, practices and lives in adult education and learning: Proceedings of the 10th ESREA triennial conference* (pp. 243-248). Zenodo. 10.5281/ZENODO.8017865

[CR73] Lim, A., & Nicolaides, A. (2024). Becoming-with-AI: Rethinking professional knowledge through generative knowing. *Reflective Practice,**25*(3), 391-405. 10.1080/14623943.2024.2321227

[CR38] Mackenzie, T., Salgado, L., Bhaduri, S., Kuketz, V., Savoia, S., & Virguez, L. (2024). Beyond the algorithm: Empowering AI practitioners through liberal education. In *2024 ASEE annual conference & exposition proceedings*, 46648. 10.18260/1-2—46648.

[CR39] Martin, D. A., Conlon, E., & Bowe, B. (2021). A multi-level review of engineering ethics education: Towards a socio-technical orientation of engineering education for ethics. *Science and Engineering Ethics,**27*(5), 60. 10.1007/s11948-021-00333-6 34427811 10.1007/s11948-021-00333-6 PMC8384818

[CR40] Mitcham, C. (2009). A historico-ethical perspective on engineering education: From use and convenience to policy engagement. *Engineering Studies,**1*(1), 35–53. 10.1080/19378620902725166

[CR41] Nair, I., & Bulleit, W. M. (2020). Pragmatism and care in engineering ethics. *Science and Engineering Ethics,**26*(1), 65–87. 10.1007/s11948-018-0080-y 30617665 10.1007/s11948-018-0080-y

[CR42] National Research Council (Ed.). (2004). *How people learn: Brain, mind, experience, and school* (Expanded edn., 9. print). National Academy Press.

[CR43] Nolan, C. (Director). (2014). *Interstellar* [Film]. Paramount Pictures (United States and Canada) Warner Bros. Pictures (International).

[CR44] Pendleton-Jullian, A. M., & Brown, J. S. (2018). *Design unbound: Designing for emergence in a white water world*. The MIT Press.

[CR45] Picon, A. (2004). Engineers and engineering history: Problems and perspectives. *History and Technology,**20*(4), 421–436. 10.1080/0734151042000304367

[CR46] Puig de La Bellacasa, M. (2017). *Matters of care: Speculative ethics in more than human worlds*. University of Minnesota press.

[CR47] Riley, D. (2003). Employing liberative pedagogies in engineering education. *Journal of Women and Minorities in Science and Engineering,**9*(2), 137–158. 10.1615/JWomenMinorScienEng.v9.i2.20

[CR48] Riley, D. (2013). Hidden in plain view: Feminists doing engineering ethics, engineers doing feminist ethics. *Science and Engineering Ethics,**19*(1), 189–206. 10.1007/s11948-011-9320-0 22033855 10.1007/s11948-011-9320-0

[CR49] Ritt, M. (Director). (1979). *Norma Rae* [Film]. 20th Century-Fox.

[CR50] Rosenberger, R., & Verbeek, P. P. (2015). A field guide to postphenomenology. In *Postphenomenological investigations: Essays on human-technology relations* (pp. 9–41). Lexington Books. https://research.utwente.nl/en/publications/a-field-guide-to-postphenomenology

[CR51] Rosenberger, R. (2023). On variational cross-examination: A method for postphenomenological multistability. *AI & Society,**38*(6), 2229–2242. 10.1007/s00146-020-01050-7

[CR52] Ryan, M.-L. (2006). From parallel universes to possible worlds: Ontological pluralism in physics, narratology, and narrative. *Poetics Today,**27*(4), 633–674. 10.1215/03335372-2006-006

[CR53] Sætra, H. S. (2019). When nudge comes to shove: Liberty and nudging in the era of big data. *Technology in Society,**59*, 101130. 10.1016/j.techsoc.2019.04.006

[CR54] Schön, D. A. (1992). The theory of inquiry: Dewey’s legacy to education. *Curriculum Inquiry,**22*(2), 119–139. 10.2307/1180029

[CR55] Secules, S. (2019). Making the familiar strange: An ethnographic scholarship of integration contextualizing engineering educational culture as masculine and competitive. *Engineering Studies,**11*(3), 196–216. 10.1080/19378629.2019.1663200

[CR56] Smith, J., Lucena, J., Rivera, A., Phelan, T., & Smits, K. (2021). Developing global sociotechnical competency through humanitarian engineering: A comparison of in-person and virtual international project experiences. *Journal of International Engineering Education*. 10.23860/jiee.2021.03.01.05

[CR57] Sparrow, R. (2020). Robotics has a race problem. *Science, Technology, & Human Values,**45*(3), 538–560. 10.1177/0162243919862862

[CR58] Stanton, A. (Director). (2008). *Wall-E* [Film]. Walt Disney Studios Motion Pictures[a.

[CR59] Suchman, L. (2007). Feminist STS and the sciences of the artificial. In *New handbook of science and technology studies* (3rd edn.). MIT Press.

[CR77] Suchman, L. (2023). The uncontroversial ‘thingness’ of AI. *Big Data & Society*, *10*(2). 10.1177/20539517231206794

[CR60] Taylor, V. F., & Provitera, M. J. (2011). Teaching labor relations with Norma Rae. *Journal of Management Education,**35*(5), 749–766. 10.1177/1052562910383967

[CR61] Teays, W. (2017). Show me a class that's got a good movie show me. *Teaching Ethics,**17*(1), 115–126. 10.5840/tej20176644

[CR62] van Manen, M. (2017). Phenomenology in its original sense. *Qualitative Health Research,**27*(6), 810–825. 10.1177/1049732317699381 28682720 10.1177/1049732317699381

[CR63] Verbeek, P.-P. (2005). *What things do: Philosophical reflections on technology, agency, and design*. Pennsylvania State University Press.

[CR64] Verbeek, P.-P. (2008). Cyborg intentionality: Rethinking the phenomenology of human–technology relations. *Phenomenology and the Cognitive Sciences,**7*(3), 387–395. 10.1007/s11097-008-9099-x

[CR65] Verschraegen, G., Segaert, B., Vandermoere, F., & Braeckmans, L. (Eds.). (2017). *Imagined futures in science, technology and society*. Routledge, Taylor & Francis group.

[CR66] Whedon, J. (Director). (2015). *Avengers: Age of ultron* [Film]. Walt Disney Studios Motion Pictures.

[CR67] Yee, S. (2017). “You bet she can fuck” – trends in female AI narratives within mainstream cinema: Ex Machina and her. *Ekphrasis Images Cinema Theory Media,**17*(1), 85–98. 10.24193/ekphrasis.17.6

[CR68] Yeung, K. (2017). ‘Hypernudge’: Big data as a mode of regulation by design. *Information, Communication & Society,**20*(1), 118–136. 10.1080/1369118X.2016.1186713

[CR69] Yorks, L., & Kasl, E. (2002). Learning from the inquiries: Lessons for using collaborative inquiry as an adult learning strategy. *New Directions for Adult and Continuing Education,**2002*(94), 93–104. 10.1002/ace.63

[CR72] Yang, X., Lim, A., Nicolaides, A., & Morkos, B. (2022). Towards the understanding of nudging strategies in cyber-physical-social system in manufacturing environments. In *ASME 2022 international design engineering technical conferences and computers and information in engineering conference*, November 11. 10.1115/DETC2022-90863

